# Dental Physiology

**Published:** 1873-10

**Authors:** Henry S. Chase

**Affiliations:** St. Louis.


### SELECTED ARTICLES.

---

### ARTICLE IV.

### *Dental Physiology.*—Concluded.

BY HENRY S. CHASE, ST. LOUIS.

I have sufficiently spoken of *natural* food. I repudiate all inorganic chemical food from the laboratory of the chemist; it is a fallacy to suppose that *artificial* lime salts will produce the desired results. Only so far as they may act medicinally to improve digestion and assimilation will they be of benefit. Nature will reject them as *factors* of nutrition, as the urine and fæces will show.

That particle of phosphate of lime which is intended to become a portion of bone or tooth must first have received a certain degree of vitality or life by the wonderful force of the vegetable cell before it can become a living portion of the animal cell. No! follow nature! give the food which she so bountifully provides for her children, and she will respond to your endeavors by granting your earnest desires.

The custom of feeding babes with starchy and gummy substances is both unscientific and practically injurious. Starch produces fat; gum is not digested at all.

It is the *fashion* to fatten babies, mothers undoubtedly thinking it a true indication of perfect nutrition. This is a great error. True health consists in the normal performance of every function of the body, and this can take place quite as successfully with a small amount of adipose substance as with the system burdened with it. Indeed excess of fat is not normal but rather pathological.

Children should not be *fed* at all, if possible, until nature has given a hint for additional food by the eruption of the incisor teeth. Then lean meat is preferable to vegetable food, and will be found of easier digestion.

The eruption of the milk teeth is a period full of dangers to the health and life of the child in civilized life. Leaving out the catalogue of contagious diseases, nearly every other ailment may truthfully be laid to the violated laws of hygiene; or the inherited latent diseases of scrofula and syphilis. The ignorance of parents on this subject is terrible; causing a destruction of life which is painful to contemplate.

The eruption of the teeth being a physiological process as innocent digestion, should not bear the obloquy which is so universally and erroneously attached to it. I cannot discuss this subject upon which I feel so much interest, because it would carry me far beyond the limits which I had prescribed in this paper, and I will therefore only say that I believe that almost every one who will give enlightened thought carefully and patiently to this question will come to similar conclusions with myself.

The violated laws of infantile hygiene, and the diseases which are forced upon the innocent being by the sins of others, retard and complicate the process of eruption to an alarming extent. Extending as it does over so many months, with the teeth in such variety of stages of progress towards growth and dentification, and absorption of the gums to admit of the passage of the crown, these diseases are constantly interfering with the beautiful cellular changes which are necessary to carry them all through to perfection. And it is indeed wonderful, and a cause for our hearts to swell with gratitude to our Creator that He has made nature so persistent, so patient in endeavoring to accomplish her good work, notwithstanding the ignorant and provoking interferences of vain man.

The treatment of infantile diseases then should be entered upon with the understanding that the eruption of the teeth is not the *cause* of any of them ; and that the welfare of the dental organs imperatively demands that all pathological conditions of the body should be as quickly removed as possible, that nature may be able to continue her wonderful work of dentification and eruption.

In regard to the process of eruption itself there is danger of too much meddling. We are too apt to think that nature is incompetent for the proper performance of her work, and therefore, many of us cut the gums of the little innocents at the beek of any ignorant mother or nurse in charge, without regard to condition.

The gums ought not to be cut, whether sick or well, unless there is distinct swelling or inflammation. If it is for the liberations of a tooth we must be sure that it has sufficiently advanced and that there is an upward pressure which cutting will relieve. If to relieve congestion, only the mucous membrane or the gums should be cut. Deep cutting is not only dangerous to the mechanical texture of the tooth, but also to life. Fatal hemmorrhage has often been the result of deep incisions. The supply arteries are large in proportion to the tissues which they are so busy now in feeding, for where there is so much to build there must be abundance of material.

If there are any who consider that infantile diaease is a subject beyond the limits of the dentists sphere, I beg them to remember that nothing is beyond the legitimate sphere of an *educated* and *intelligent* dentist, who desires to see the beautiful works of God restored to that place in the temple of nature where He first placed them in His infinite love. To this end let us all strive until we shall no more hear the impious remark, so often made in our offices, "Why did not God make the teeth to last as long as our lives?"

Eruption. This physiological process occupies from about the seventh to the thirtieth month for the fisst or milk teeth This is certainly a very interesting and important period in the child's life. The mortality of children at this time of life is enormously out of all proportion to other periods, and is indeed frightful to contemplate. My views in regard to the dependence of infantile diseases upon the process of eruption have already been expressed. Hygienic measures of the very best kind should be adopted to bring the child of civilization, and perhaps the heir of hereditary diseases inimical to the highest attainment of bodily perfection, into such relations with natural laws, as will best neutralize all those untoward influences which render the thread of an infant's life brittle and uncertain.

The mother's milk is one of the most important things to be looked after, and one, too, almost entirely neglected. It is impossible to make good milk out of poor material. But my previous remarks on this subject will suffice. Still good materials may be spoiled by mixture with those which are deleterious. Although a mother may have supplied her blood with the best materials for the growth of the body in general, and the teeth particular, yet she may partake in condiments, relishes and drinks which will disagree with the stomach of her child, and render it unable to digest the food she offers it. Even if digested and taken into the circulation by the infant they often cause an irritation in the histological tissues which result in disease. Ohions and other food of that character cause vomiting of the child. Pickles and other acids cause griping pains and diarrhœa. Coffee produces vomiting, bilious colic, and great irritability of the nervous system. Tea and coffee cause great wakefulness. Under excitement of the mind and passions have often killed infants as certainly as strychnine. Over-work, and heating the blood higher than normal, are very injurious to the health of the child. Medicines also partaken of by the mother, are very likely to produce pathological effects on the nursing child. Surely there is nothing strange in the fact that so many die in infancy, when the laws of nature are so constantly violated and the little innocent placed in such inharmonious relations to physiological laws.

If a mother cannot furnish *pure* and nutritious food for-her child, a wet nurse should be had if possible, who can do so, and not without.

Whatever best promotes the *general health* of the child will accord with the best health of its teeth. Therefore, dress, bathing, exercise, fresh air and the sun's rays must all be considered and appropriately applied for its benefit. A lengthened discussion of these topics in this paper would be inappropriate.

The first teeth seldom erupt irregularly, or rather they present to the eye when all *are* erupted that beauty and regularity which is so delightful to the mother, and the earnest physiologist.

There are tardy eruptions and premature eruptions of the first teeth. In extreme cases both conditions are symptomatic of an impaired constitution, or previous arrest of development under influences which prevented nutrition. For it is generally the case that teeth erupted at birth or soon after, will be found devoid of roots in normal length. Such facts should lead the medical or dental attendant to enquire into the present and past hygienic habits of the mother, and correct them if necessary. Placing mother and child in the best physiological conditions, by whatever means are necessary, will be the best guarantee that nature will carry out her intentions in the perfect eruptions of the child's denture.

It is never too early to begin the practice of cleansing the infant's teeth. Water is very grateful to most children when cutting the teeth, and the teeth should be bathed and cleansed with pure water after the act of suckling.

If it is said that this is unnecessary, and that animals and many mothers also, never do this, and still the teeth of their offspring are sound, I merely reply, that while the habits of mothers are artificial, and their present constitutions the result of artificial habits also, we must use artificial means to restore or promote the health of themselves and their offspring. Thousands of children loose their incisors before they are three years old by decay. This shows imperfect dentification and calcification of the teeth, but who can say that cleanliness of these organs during the period of lactation would not have prevented their chemical erosion by the acids formed by the putrefaction of the milk adhering to them, and the abnormally acid epithelial and glandular secretions.

The mothers milk has in many instances, a tendency to rapidly sour and disorganize, causing not only gastric and other constitutional derangements in the child, but also local lesions in the mouth.

The time of weaning a child must depend on the health of the mother and child. About the fifteenth month is perhaps the best, although it is better to vary the length of suckling so as to have the weaning in cool weather. In fact it is better that suckling should continue through the *second summer* if possible. The health of the child is thereby more fully secured, as gastric derangements are apt to ensue if the child is dependent on other food than mother's milk the second summer. For at this time it has not erupted the molars, and mastication is yet imperfectly performed at the very time when it should be thoroughly done if done at all.

Before weaning time has arrived the milk of the cow should be used in addition to that of the mother to some extent. Lean meats finely cut, and broths of meat may also be used advantageously, if judiciously employed. Very little vegetable food should be used before the child has cut its grinders. Starchy substances, such as corn starch, and fine flour bread, arrow root, &c., ought not to be used as food for the infant. Even if tolerated by the organism it is not natural, and foundations will be laid for gastric disorders which will more than likely take place in a year or two later under such a regime. Wheat finely ground but unbolted and divested of the course bran, if moderately used before weaning, at the latter part of lactation, will give the child the best pabulum for blood formations and lay the foundations for vigorous digestive organs.

A sudden transition from the mother's milk alone to other food is very likely to be disastrous in proportion to its departure from the proximate elements of the material milk.

Children should be taught to thoroughly masticate their food. The eruptions of the grinders may serve as a hint for food which *requires* mastication to make it comfortable to swallow. The teeth absolutely require exercise for their best health, and food should not be given to children at this age that does not require it. One very obvious reason for the perfect teeth of savages and semi-barbarous people is found in the fact that everything they eat requires mastication to make it even comfortabale to swallow, leaving out the heightened pleasure experienced by thorough comminution of food and its mixture with saliva. Mastication gives strength and vigor to the roots and delicate membrane investing them, and cleanses the teeth by attrition. The gums are also benefitted, and by continuity of tissue the other parts of the mouth and fauces.

By the end of the third year all the milk teeth are erupted, in good *position* generally. They ought also to be free from decay if decent hygienic measures have been heretofore in operation. The child is now old enough to use a soft tooth brush and should be taught to use it after every meal and lunch. They may very properly be allowed to eat five times a day, but not oftener. A child who is always eating will be sure to have dirty teeth, which will soon result in erosions and decay, besides a sour and weak stomach.

Sweets ought not to be forbidden in moderate quantity. There is a natural craving in infants for saccharrine substances; and there is no good physiological reason why they should not be allowed temperately. It is much better, however to give a child white lump sugar than candies which often contain noxious ingredients. Sugar as sugar never injures the teeth; it is only when converted into acid that it acts injuriously. Neither will ripe fruit containing considerable acid injure the child's teeth when moderation is used in their employment.

The teeth of the child from their first eruptions ought to be under professional supervision, but at any rate it ought not to be deferred later than the third year.

At this point I will leave the subject, for I see before me a vast field for thought, which I must not now enter, as it would lead me far beyond the limits of your patience, namely, the preservation of the milk teeth until replaced by the permanent ones, and the care of the permanent teeth during the process of replacement.—*Missouri Den. Journal.*

